# Novel Genes from Formation to Function

**DOI:** 10.1155/2012/821645

**Published:** 2012-07-03

**Authors:** Rita Ponce, Lene Martinsen, Luís M. Vicente, Daniel L. Hartl

**Affiliations:** ^1^Centro de Biologia Ambiental (CBA), Faculdade de Ciências da Universidade de Lisboa, 1749 Lisboa, Portugal; ^2^Centre for Ecological and Evolutionary Synthesis (CEES), Department of Biology, University of Oslo, Blindern, 0316 Oslo, Norway; ^3^Department of Organismic and Evolutionary Biology, Harvard University, 16 Divinity Avenue, Cambridge, MA 02138, USA; ^4^Centre for Environmental and Marine Studies (CESAM), Faculdade de Ciências da Universidade de Lisboa, 1749 Lisboa, Portugal

## Abstract

The study of the evolution of novel genes generally focuses on the formation of new coding sequences. However, equally important in the evolution of novel functional genes are the formation of regulatory regions that allow the expression of the genes and the effects of the new genes in the organism as well. Herein, we discuss the current knowledge on the evolution of novel functional genes, and we examine in more detail the youngest genes discovered. We examine the existing data on a very recent and rapidly evolving cluster of duplicated genes, the *Sdic* gene cluster. This cluster of genes is an excellent model for the evolution of novel genes, as it is very recent and may still be in the process of evolving.

## 1. Introduction

The availability of complete genome sequences allows the comparison of genomes, thus revealing the differences in gene complement and demonstrating the nature of the changes that occur in the evolution of genomes. In particular, genomic analysis showed that more than one-third of the eukaryotic genome is composed of gene duplications and gene families (e.g., [1–4]), highlighting the prominence of duplications in the evolution of genomes. The study of whole genomes also allows an analysis of the rates and dynamics of duplications as well as the divergence and silencing of duplicates (e.g., [5–7]). Moreover, evidence indicates that the rates of duplication are extremely high and of the same order of magnitude as the rate of mutation per nucleotide; for example, the frequency of duplication for coding genes was found to be 0.01 per gene per million years [8] and for internal duplications of gene segments, 0.001–0.013 duplications/gene per million years [9]. 

The fates of gene duplicates can be very different according to the mutations they undergo and the selective pressures they are under. After a gene is duplicated, the most common fate seems to be the loss of function of one copy by the acquisition of degenerative mutations, while the other copy retains the original function. The originally identical copies can also both be maintained in the genome, allowing a higher production of the corresponding RNA or protein. Subsequent mutation by retrotransposon insertion into one of the copies can affect adaptive evolution because the effect on the phenotype can in some cases be beneficial to the organism [10]. Partial gene duplications can also occur, and if the duplicated part involves a structural or functional domain the new gene can increase the functional complexity of the encoded protein. It has also been proposed that if two copies of a gene acquire mutations in distinct subfunctions, the copies may undergo complementary loss of function in such a way that both copies are required to perform the original function; both copies would be maintained by selection [11]. Probably the most infrequent fate for a gene duplicate, and the one with most evolutionary impact, is for a duplicate copy to gain a new function by acquisition of mutations and to be maintained subsequently by selection while the old copy retains the original function. This last phenomenon leads to novel cellular functions and is the basis of the formation of divergent gene families. For a recent review of models of the evolution of gene duplications and the predictions for each model, see Innan and Kondrashov [12]. 

Gene duplication has long been thought to be a primary source of material for the evolution of new genes and new functions. The potential of gene duplication was first recognized by Haldane [13], Bridges [14] and Muller [15]. In 1970, these ideas resurfaced with Ohno [16] who maintained that new genes and novel functions arise only by duplication and who presented evidence for the role of genome duplications in the evolution of multicellular organisms. Since then, the importance of duplications and of the divergence of duplicated copies has been widely supported (for a review see, e.g., [17–20]).

However, other evidence has accumulated showing that there are also other mechanisms responsable for the evolution of novel functions, such as exon shuffling, retroposition, gene fusion, and gene fission, and that several mechanisms can act together ([21–23] and recently reviewed in Kaessmann [24]). Another issue that has received attention is the formation of new regulatory regions, as these can be responsible for the development of novel expression patterns (for review see, e.g., [25–28]). In this paper, we will focus on the origin of very recent genes, which are of special interest since they may have not yet lost the signature of the events that took place during their formation, therefore conveying valuable information about those events.

## 2. Mechanisms of Origin of Novel Genes 

The origin of novel genes has been extensively studied. In Table 1 is summarized the information on a sample of novel genes that reveals a picture of the events involved in their origin and their function. The sample is limited to genes that originated less than 50 million years ago to avoid confounding events of origination with effects of later evolution; this cutoff is also based on the dynamics of formation, preservation, and decay of duplicates and chimeras found by Rogers et al. [29]. Many of the novel genes described and characterized are from *Drosophila* species. The extensive work carried out on the evolution of new genes in *Drosophila* (see e.g., [30]) reflects the fact that *D. melanogaster* is a well-studied model organism at the molecular level that also has a long history of evolutionary studies. 

Overall, there is no single mechanism of the molecular events involved in the formation and maintenance of novel genes, but rather several mechanisms. While gene duplication is conceded to have an important role in creating genetic novelties, an alternative mechanism for the origin of new genes is the shuffling of existing genes or functions to form chimeric genes. However, many chimeric genes derive from previously duplicated material, as shown by the presence of the parental genes in the genome and the organization of the chimeras [29, 31]. A chimeric gene would hardly be recognized as such were the parental genes not present. A second type of chimeric gene arises not from tandem duplication, but from duplication through retrotransposition. In addition, it has been shown that both nonhomologous recombination and nonallelic homologous recombination can lead to the formation of chimeric genes which both can be facilitated by repetitive elements (transposable elements or satellite DNA sequences) [32]. Yang et al. [32] identified 17 chimeric genes formed by ectopic recombination within the last 12 Mys in the *Drosophila melanogaster*.

The contribution of chimeric genes to the evolution of genetic novelties has been revealed by recent studies. Rogers and colleagues [29, 31] compare the fate of simple genetic duplicates versus genetic duplicates that underwent fusion to form chimeras in *D. melanogaster*. The results are very interesting: gene duplications are formed at a rate of about 80.4 duplicates per million years, but only 4.1% are preserved; chimeras are formed at a rate of about 11.4 per million years and show a similar rate of decay (with 1.4% preservation) [29]. Some chimeric genes were also implicated in selective sweeps [31], revealing the impact of this kind of molecular event. This analysis of the *D. melanogaster* genome is interesting, but raises the question whether the same pattern would be observed in species that do not have such a high rate of DNA loss as *D. melanogaster* as found by Petrov and colleagues [33–35].

Equally relevant, and conceptually on the opposite side of the spectrum, is the origination of new genes “from scratch” or *de novo*, the case when coding sequences are derived from noncoding DNA, for example, introns and other untranslated regions [36–39]. This reutilization of noncoding sequences may have important consequences in the fate of such genes. 

## 3. Tissues Where Novel Genes Are Expressed 

Among the examples of new genes with very recent origin, many are involved in male reproduction, in particular in spermatogenesis. It is the case of *Jingwei*, which appears to be expressed in the testis [43]; *Odysseus*, which contains rapidly evolving homeodomains involved in sperm function [56]; *Dntf-2r*, which has male-specific expression [50]; *K81*, which is expressed in primary spermatocytes [58]; *Sdic*, whose product is incorporated into the sperm tail [47]. The prominence of expression in male reproductive tissue has led to the hypothesis that spermatogenesis is more prone to the cooption of novel genes and functions, while other developmental processes may be under a higher level of selective constraint [63]. As debated in Nielsen et al. [64], the testes undergo intense selection pressures because the cells are subject to genetic conflict, sperm competition, reproductive isolation and are exposed to germline pathogens and mutations that cause segregation distortion. An interesting observation in this regard is that newly retroposed duplicated genes in *Drosophila* are dominated by genes going from the X chromosome to the autosomes, not the opposite direction, and most of these have evolved a testis-specific expression pattern [65]. A possible explanation is that this predominantly one-way pattern is driven by positive selection because the retroposed genes escape X inactivation during spermatogenesis and are therefore free to develop expression patterns in the testis [65].

Kaessmann [24] emphasizes the importance of transcription during spermatogenesis, during which a permissive state of the chromatin may allow a widespread transcription of genes that would not otherwise be expressed. 

Additionally, during spermatogenesis, there are numerous cell divisons, affording an opportunity for new mutations to arise, and those may affect sperm performance. Hence, spermatogenesis offers a large arena of competition in which cells and their descendants are under intensive selective pressure. 

An important pattern observed in speciation, often considered the first step in the evolution of complete sterility and inviability, is Haldane's rule, which states that, in an interspecific cross, if one sex is sterile or inviable, this sex is the heterogametic sex [66]. If novel genes appear more often in male reproductive tissue than in female reproductive tissue—as observed in the summary of novel genes in Table 1—this could be an explanation for Haldane's rule when the heterogametic sterile F_1_ hybrid is the male, but not in the cases when it is the female. In *Xenopus*, in which males are the homogametic sex, males are always sterile in interspecific crosses [67]. However, while *Xenopus* seems an exception to Haldane's rule, it may be a consequence of the fast evolution of male-specific genes. Strong sexual selection in males is reflected by rapid evolution of genes in male reproductive tissue, which then may cause hybrid sterility in recently diverged sister species even when males are homogametic. From this angle, tissue specificity of novel genes seems more important to hybrid sterility than heterogamy. It would be interesting to examine the tissue specificity of novel genes in species in which the female is the heterogametic sex, but this is beyond the scope of our paper. 

## 4. The *Sdic* Gene Cluster

The *Sdic* gene is a recently evolved chimeric gene in *D. melanogaster*, discovered and described by Nurminsky and colleagues in 1998 [47, 68]. This gene possesses several unique features that provide an exceptional opportunity for the study of new gene functions, the fate of gene duplications, and the evolution of male reproductive traits. 

**Figure 1:**
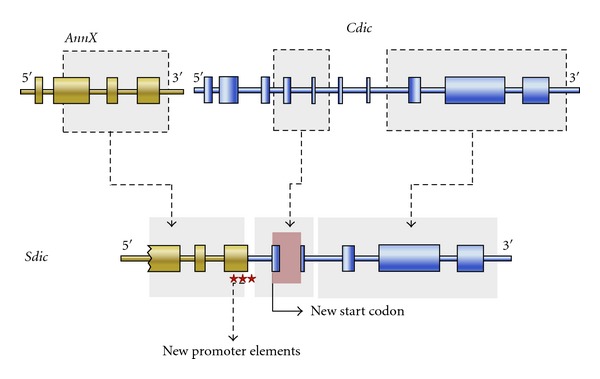
Formation of the *Sdic* gene from parts of the genes *AnnX* and *Cdic*. Introns are represented as thin cylinders and exons as thick cylinders. The stars represent *Sdic* promoter elements.

Sequence analysis of the *Sdic* gene revealed that *Sdic* is a chimera of two genes that exist intact in the genome. *Sdic* is composed of parts of *AnnX*, which encodes an annexin protein, and *Cdic *(also referred to in the literature and FlyBase as *sw*), which encodes an intermediate polypeptide chain for the cytoplasmic dyneins [47]. The structure of *Sdic* along with the fact that *AnnX* and *Cdic* exist intact in the genome indicates that *Sdic* originated as a duplication and fusion of *AnnX* and *Cdic*, followed by small deletions and rearrangements. Its formation involved the creation of novel promoter elements (which provided testis-specific expression) from the fusion of portions of an *AnnX* exon and a *Cdic* intron. Its coding region, however, derived solely from *Cdic*. The comparison of the coding region of *Sdic* with *Cdic* shows that *Sdic *lacks the 3′ region of *Cdic* (which corresponds to 100 amino acids residues at the C-terminal part of the *Cdic* protein) and at its 5′ end underwent extensive refashioning by the occurrence of multiple mutations, deletions (including frameshift deletions), and insertions, culminating in a new 5′ exon that encodes a totally novel N-terminus for the protein [47, 69] (Figure 1).

**Figure 2:**
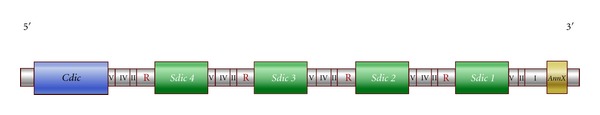
The *Sdic* gene cluster. The cluster is composed by four *Sdic* genes, with an *RT1C* retrotransposable element upstream of each Sdic gene. The cluster is located between the parental genes *Cdic* and *AnnX*. *Cdic* is represented in blue, *Sdic* genes in green and *AnnX* in yellow; intergenic regions are grey; R represents *RT1C* elements. These genes are located in the minus strand, so the order of genes in this figure is the opposite order of these genes in Flybase.

There are several copies of *Sdic* located in tandem at the base of the X chromosome, in region 19 of the larval salivary gland polytene chromosomes, forming a gene cluster. This repeated region is flanked by the parental genes, on the 5′ side by *Cdic* and on the 3′ side by *AnnX*. According to the available genomic sequence of *D. melanogaster, the Sdic* gene is repeated four times in tandem between the genes *Cdic* and *AnnX* genes. Within this cluster there are also four dead-on-arrival retrotransposable elements of the *RT1C* family, one *RT1C* copy located upstream of each *Sdic* gene copy [70] (Figure 2).

*Sdic* is present in all wild-type strains of *D. melanogaster* [47], but it has not been found in any other species of the *D. melanogaster* subgroup. In the species closest to *D. melanogaster* (*D. simulans*, *D. mauritiana*, *D. yakuba*, *D. teissieri*, and *D. erecta*) *AnnX* and *Cdic* are adjacent to each other, without signs of an ancestral *Sdic* gene or *RT1C* element between them [71]. Furthermore, in these species, *AnnX* and *Cdic* do not show any signs of duplication and are reasonably conserved across species [71]. Consequently, the formation of the *Sdic* gene and the subsequent duplication that formed the entire cluster happened only in the lineage that gave rise to *D. melanogaster*; the original *Sdic* and the *Sdic* cluster were formed within the last 2 million years, after the split of the lineage that formed *D. melanogaster *and its sibling species *D. simulans*.

A model for the evolution of this cluster was developed based on the available genomic data [70], and the divergence of the *RT1C* copies, which are expected to be evolving neutrally, was used to date the duplications [48]. In the ancestral situation *AnnX* and *Cdic* must have been adjacent to each other. An initial event duplicated *AnnX* and *Cdic*, and then one or more deletions fused one copy of *AnnX* and one copy of *Cdic* giving rise to an ancestral *Sdic*. During the early steps of the formation of the ancestral *Sdic*, a dead-on-arrival retrotransposable element from the *RT1C* family was inserted upstream of the ancestral *Sdic*. The ancestral *Sdic* and its upstream region were duplicated in the last 232–463 thousand years, giving rise to two *Sdic* genes (and two *RT1C*, one upstream of each *Sdic*). Another duplication in the last 100–180 thousand years gave rise to four *Sdic* genes (and four *RT1C*, one upstream of each *Sdic *copy).

While the *Sdic* cluster has been isolated in the extremities of BAC clones, it is difficult to determine the overlap of the BAC sequences; moreover, there are nonassembled pieces of BAC clones containing *Sdic* portions that do not seem to match any of the assembled copies. The recent *in situ* hybridization work by Yeh et al. [49] indicates that all *Sdic* copies should be in region 19 of chromosome X. Given the young age of the cluster, the duplicates are predicted to be very recent and to have few or no differences in sequence, making it extremely difficult to confirm the exact number of genes by present sequencing and assembly techniques. The available sequences of assembled and nonassembled BAC clones could be explained either by four copies in tandem or eight copies that resulted from a duplication of preexisting four, yielding eight with the same pattern of similarities. The hypothesis of a cluster formed of four copies is the most parsimonious; however eight copies in tandem would be closer to the original Southern blot experimental results by Nurminsky et al. [47], which suggested as many as 10 duplicates. 

One question still unanswered is that of the expression of the genes that belong to this cluster. Although the cluster is composed of four almost identical *Sdic* genes, there is no evidence for expression of all copies. The RNA sequences described in Nurminsky et al. [47] and Kulathinal et al. [63] are similar to what would be the expected transcription of *Sdic1*. Later work found evidence of expression for both *Sdic1* and *Sdic3* [49, 72]. *Sdic1* is the oldest gene in the array and *Sdic3* was originated from a duplication of *Sdic1*, being the second oldest. The promoter sequences of all copies are identical, except for the promoter of *Sdic1*, which differs from the others by two nucleotides. Although these changes at the promoter level might not be relevant, there is experimental evidence that the promoter of *Sdic1*, at least, is a functional promoter: Nurminsky et al. [47] tested the *Sdic1* promoter *in vivo* and *in vitro* and found it to be functional.

The presence of extra genetic material in the *Sdic *region when one compares *D. melanogaster *with the other species of the *D. melanogaster* subgroup, extra material that contains a cluster of nearby identical genes and dead-on-arrival transposable elements is remarkable. *D. melanogaster *has a high rate of gene loss of unessential genetic material caused by deletions [33–35]. Additionally, according to existing models of the fate of gene duplicates and in light of the high rate of nonessential DNA loss in *D. melanogaster*, if not all copies of *Sdic* are functional then one would predict that the extra copies of *Sdic* would be in the process of degeneration showing many deletions and mutations in the coding sequences as well as the *RT1C* elements. However, the *Sdic* copies and *RT1C* elements appear very similar to each other. It is possible that, given the young age of the cluster, degeneration is in such early stages that such mutations have not occurred or that those that may have occurred have been repaired by gene conversion from nonmutated copies. It is also possible that the *Sdic* cluster has important cellular functions yet to be discovered. Novel gene functions are frequently associated with rapid changes and show signs of positive selection [44, 50, 73], supporting the idea that novel genetic functions allow adaptive changes. There is limited evidence for positive selection of *Sdic* and at least one selective sweep in this region [47, 63, 74]. 

A key question concerning any novel gene and the functional role it fulfills is whether the function itself is novel or whether the function is redundant or overlapping with an already existing functional gene or genes. The function of *Sdic* protein was deduced from its sequence to be a sperm-specific dynein intermediate chain, and this deduction was supported by the finding of the *Sdic1* gene product in the sperm tail [47]. Is the Sdic protein redundant with another sperm dynein intermediate chain or has it supplanted a preexisting gene? Has *Sdic* played a role in the speciation events in the split of the lineage of *D. melanogaster *from its sibling species and has *Sdic* had an effect in adaptation or sexual selection in *D. melanogaster*? 

These questions on the role of *Sdic* have been addressed very recently in experiments in which Yeh and colleagues [49] knocked out the *Sdic* region in *D. melanogaster* and looked for phenotypic effects on male reproduction. While no effects were detected in progeny size or sex ratio, males without *Sdic* had sperm that were less competitive in the female reproductive tract in being more easily displaced by sperm from subsequent males and, to a lesser extent, in being less able to displace sperm from previous males. These results support the role of the *Sdic* protein in sperm competition, but most importantly they answer the central question for new genes of “how did the sibling species manage without this function?” the work by Yeh et al. [49] shows that *Sdic* plays an important role in reproduction, and although it is not an essential gene, it gives an advantage in sperm competition, which could in turn be related to the positive selection. 

It remains to be determined whether positive selection has acted upon all copies of *Sdic *and if the copies are subjected to the same selective pressures, but the importance of this gene cluster in reproduction has at least been demonstrated, and the results may help to shed light on the general issue of the function of novel genes

## 5. Concluding Remarks

Ultimately, differences observed between species are due to differences at the genome level. Genomic studies are revealing the extent of these differences—in gene number, in encoded functions, in expression—and are also revealing the mechanisms involved in the evolution of genomes. The analysis of particular newly evolved genes provides information in finer detail, which hopefully can be generalized and help to understand the evolution of new genes and new functions. Equally as important as the formation of new coding sequences is the formation of regulatory regions responsible for new patterns of expression as well as the processes leading to spread and maintenance of the novel gene in the population. 

Bacterial genome studies have made very clear that, at least in bacterial species, a great part of the genes are not shared by all individuals of a species [75]. Different strains of the same species share a core genome containing genes present in all strains; however there is also a pan-genome consisting of genes present in only a subset of strains. As more complete genome sequences become available, we will be able to determine if similar patterns are observed in eukaryotes.

## Figures and Tables

**Table 1:** Novel genes described (less than 50 million years (mys) old): Their formation, expression and function. The genes are ordered by age.

Gene	Mechanisms	Functions	Expression tissue	Species, genome location	Age (mys)	References
*Monkey-king (mkg)*	Duplication by retroposition and gene fission	Not determined	*D. simulans/D. sechellia *only males, *D. mauritania* males and females	*D. simulans, D. sechellia, D. mauritania, X* chromosome (chr.)	1-2	[23]

*Quetzalcoatl*	Duplication, gene fusion	Not determined	Early pupae, adult females, and male testes	*D. melanogaster*	<2	[40]

*Ifc-2h*	Duplication by retroposition	Not determined	Whole body	*D. simulans, D. sechellia, D. mauritiana*	2	[41]

*Hun*	Exon shuffling by illegitimate recombination	Not determined	Testis	*D. sechellia, D. mauritania, D. simulans*	2-3	[42]

*Jingwei*	Exon shuffling, retroposition	Not determined	Testis	*D. teissieri, D. yakuba, D. santomea*	2-3	[43]

*Sphinx*	Exon shuffling, retroposition	Male courtship behaviour	Embryo, pupae, adult of males and females	*D. melanogaster*, *chr. 4 *	2-3	[44, 45]

*Poldi*	*De novo* emergence from intergenic DNA	Involved in spermatogenesis, perhaps through chromatin modification pathways	Testis	*Mus mus domesticus* and four other species in the *Mus* genus.	2.5-3.5	[38]

*Adh-Twain*	Duplication, gene fusion	Not determined	Larvae, adult males and females	*D. subobscura, D. guanche, D. madeirensis*	3	[22, 46]

*CG32582, CG32712, CG15323, CG32690, CG31909*	From non-coding sequences	Not determined	Testis	*D. melanogaster, D. simulans*	2–5	[37]

*Sdic*	Duplication, gene fusion	Sperm competition	Testis	*D. melanogaster*, X chr.	<5.4	[47–49]

*Dntf-2r*	Duplication by retroposition	Not determined	Testis	*D. melanogaster, D. simulans, D. sechellia, D. mauritiana, 2nd chr.*	3–12	[50]

*Nsr* (*kep1* gene family)	Duplication	Spermatogenesis	Testis (enriched in primary spermatocytes)	*D. melanogaster* subgroup, 2nd chr.	5.4–12	[51]

*CG3927 *(kep1 gene family)	Duplication	Spermatogenesis	Testis (enriched in primary spermatocytes)	*D. melanogaster* subgroup, 2nd chr.	5.4–12	[51]

CG4021 (kep1 gene family)	Duplication	Spermatogenesis	Testis (enriched in primary spermatocytes)	*D. melanogaster* subgroup, 2nd chr.	5.4–12	[51]

*Sflc*	Duplication	Male and female fertility, life expectancy	Not specified	*D. melanogaster* subgroup	6–11	[52]

*Hydra*	*De novo* emergence from intergenic DNA	Not determined	Testis	*D. melanogaster* subgroup	<13	[53]

*PIPSL*	Exon shuffling, retrotransposition	Ubiquitin binding	Testis	*Humans Chimpanzees*	15–19	[54]

*Adh-Finnegan*	Duplication, gene fusion	Not determined	Pupae and adults	*D. buzzatii, D. hydei, D. mettleri, D. mojavensis, D. mulleri* (repleta group)	20–30	[45, 46, 55]

*Odysseus (OdsH)*	Duplication	Involved in sperm function	Mainly in male reproductive tissue, but also detected in larvae and embryo	*D. melanogaster, D. simulans, D. sechellia, D. mauritiana, D. yakuba*	<40 (after split of subgenus *Sophophora* and *Drosophila *	[56, 57]

*K81*	Duplication by retroposition	Telomere capping in postmeiotic male germ cells	Male specific expression (testis: primary spermatocytes), but weak expression also in wild-type females	*D. melanogaster* subgroup	<30	[58, 59]

*Obp57d and Obp57e*	Duplication	Taste sensation of octanoic acid	Taste sensilla on the legs	*D. melanogaster* subgroup	<30	[60, 61]

*PGAM3*	Exon shuffling, retroposition	Possibly phosphoglycerate mutase activity	White blood cells	Humans, chimpanzees, macaques, *X chr*.	>25	[45, 62]
